# Medical Fees within the Profession

**Published:** 1916-04-22

**Authors:** 


### MEDICAL FEES WITHIN THE PROFESSION.

The question of professional fees is often a difficult one in medical practice. But it has to be faced, for few indeed are they who can afford to consider the matter as one of no importance. Equally few, we imagine, are the practitioners to whom the fee is the main consideration. It is one of the honourable traditions of the profession to regard the position and resources of the patient and to temper strict justice with abundant forbearance. No one would counsel otherwise. Possibly this custom is sometimes abused, and every man owes a duty to his fellows to prevent such abuse so far as prevention is possible. Yet even when these two principles are accepted the translation into practice is not easy, and if there are a few mistakes in the direction of excess there must be many on the other side.

A special aspect of the question of fees is the application of it to members of the profession. By custom and courtesy it is usual for practitioners to attend and advise fellow-practitioners and their families without charging fees. The matter is not one of right or duty, but of kindliness and good will; and manifestly it has its pleasing side. There is, however, another aspect, and this does not always receive consideration. While few practitioners would refuse to extend this courtesy to a neighbour, there are certainly some who would prefer not to receive it. The refusal is not due to any churlish reason, but to a preference for an arrangement which does not involve any sense of financial obligation. Between close personal friends such obligation may perhaps have no place, and this probably applies generally when the doctor himself is so unfortunate as to be a patient. But when members of the family are ill there are not a few practitioners who would prefer to pay fees rather than to accept gratuitous services. To a strong feeling on such a point respect ought to be paid, for no one has the right to inflict on another an unwelcome obligation. Further, it cannot be doubted that under such an arrangement there is a freedom to ask for help which can hardly exist when fees are refused. The sensitive mind shrinks from a request which is felt to be one that places the whole burden on someone else's shoulders, and this without recognition other than gratitude. Escape is sometimes attempted through the medium of a present, the choice of which may create anxiety to the giver and its arrival perplexity to the recipient. Altogether, therefore, there is something to be said for the business arrangement, while allowing that the usual custom has much grace and can often be followed with mutual advantage and good will among friends and neighbours.

A special difficulty arises when for any reason a consulting physician or surgeon is asked to see the patient. There may be personal or professional relations, or even questions of finance, which put all thoughts of fees promptly out of court. But when these do not exist there is certainly some hardship in both directions if any proposal of payment is excluded. The one man has given time and perhaps made a considerable journey, while the other hesitates to ask for a repetition of the favour, however much he may desire it. It is with no desire to limit inter-professional helpfulness that we recognise the existence of occasions on which it would be better for all concerned that a fee appropriate to the event should be paid.

